# A Statistical Mechanics Model to Decode Tissue Crosstalk During Graft Formation

**DOI:** 10.1002/advs.202523373

**Published:** 2026-01-07

**Authors:** Ang Dong, Yihan Meng, Stephen Shing‐Toung Yau, Shing‐Tung Yau, Rongling Wu

**Affiliations:** ^1^ Beijing Key Laboratory of Topological Statistics and Applications for Complex Systems Beijing Institute of Mathematical Sciences and Applications Beijing China; ^2^ Institute of Statistics and Big Data Renmin University Beijing China; ^3^ Yau Mathematical Sciences Center Tsinghua University Beijing China

**Keywords:** gene regulatory network modeling, graft incompatibility, plant grafting, tissue crosstalk, vascular reconnection

## Abstract

Grafting has been practiced for millennia to combine the best characteristics of two plants. Despite recent molecular discoveries that gain insight into plant grafting, the systematic characterization of its underlying mechanisms is still lacking. Here, we take a step toward filling this gap by developing a generalized statistical mechanics model to decode genomic crosstalk between the scion and rootstock. Instead of traditional objectives of identifying individual genes that are differentially expressed between the two organs, our model codes thousands of interactive genes into informative, dynamic, omnidirectional, and personalized networks (idopNetworks) that program and rewire scion‐rootstock crosstalk. We design an experiment of reciprocally micrografting young tissues to validate the application of idopNetworks to the genomic characterization of graft formation between two distantly related *Populus* species. Given its capacity to reveal the most comprehensive genomic underpinnings for proper interactions of the scion with rootstock to develop new plants, the idopNetworks model can be extended for the mechanistic exploration of a wide range of biological, evolutionary, and medical phenomena.

## Graphical abstract

We introduce a statistical mechanics framework to decode the genomic crosstalk governing plant grafting. By integrating evolutionary game theory with transcriptomics, we reconstruct idopNetworks (informative, dynamic, omnidirectional, and personalized networks) that map scion–rootstock interactions. This framework identifies hub genes driving vascular reconnection and compatibility, offering a unified computational strategy for dissecting complex tissue communication mechanisms.

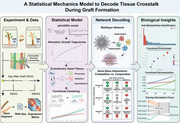

## 1 Introduction

Originally discovered in nature [1], grafting has been widely used as an asexual propagation technique to improve the yield, quality, and ornamental values of plants [2, 3]. In recent decades, grafting has also increasingly served as an approach for studying the long‐distance movement of molecules [4, 5, 6]. Grafting represents a biological process of attaching and fusing different plant segments (termed the scion and rootstock) into a chimeric individual [7, 8, 9], whose success precisely relies on the molecular mechanisms of crosstalk between these two segments [10]. Previous molecular studies strived to identify the differentiated degree of genes expressed between the scion and rootstock and predict signaling cascades and downstream biological processes underlying vascular reconnection and regeneration [2, 11]. A number of genes have been identified to mediate scion‐rootstock interactions at the graft junction [12, 13, 14, 15, 16]. For instance, in Norway spruce (*Picea abies*) grafting, *PHYTOCHROME A SIGNAL TRANSDUCTION 1* (*PAT1*) was up‐regulated to act as a core regulator of graft healing [11]. This gene was also found to be strongly up‐regulated during *Arabidopsis thaliana* grafting [17]. Early activating transcription factors, such as *ETHYLENE RESPONSE FACTORs* (*ERFs*), *DNA BINDING WITH ONE FINGER* (*DOF*), *NAC DOMAIN‐CONTAINING PROTEINs* (*ANACs*), and *WUSCHEL‐RELATED HOMEVOX* (*WOX13* and *WOX14*), are differentially expressed during grafting, playing a pivotal role in facilitating tissue adhesion, callus formation, and vascular differentiation [13, 14, 18, 19, 20].

Vascular reconnection is a complex physiological process involving a number of interactive and interdependent genes and other biological entities – proteins, metabolites, and even microbes, which transmit their signals across the scion and rootstock [12, 15, 21, 22, 23]. Alteration in gene expression, protein synthesis and metabolic activities induces the movement of biomolecules in the phloem throughout the graft union, as an essential step toward promoting scion‐rootstock communication and sustaining interfacial ecological homeostasis [24, 25]. To better explore the mechanistic basis of graft compatibility and incompatibility, a crucial issue needed to be addressed is how to characterize the roles of enormous entities as a cohesive whole to mediate graft formation. While advanced sequencing techniques have made it possible to generate a vast amount of omics data at high resolution, a powerful computational model is currently lacking to combine all grafting‐related entities into gene regulatory networks for the reconnection and regeneration of vasculature and plasmodesmata after grafting [12, 26].

We argue that the scion and rootstock interact with each other to maximize the growth of the grafted plant in a way explained by game theory [27]. To acquire such maximum growth, both the scion and rootstock act like players to strive to develop an optimal strategy in response to the strategies of their counterparts, and this process of reciprocal adaptation continues until the Nash equilibrium is reached [28]. By combining game theory and evolutionary theory, Maynard Smith and Price (1973) proposed the notion of an evolutionarily stable strategy that refines and extends the Nash equilibrium through dynamic modeling without the rationality assumption [29, 30]. Sun et al. (2021) integrated evolutionary game theory and predator‐prey theory to develop a system of mixed ordinary differential equations (mODEs) [31], which decompose the payoff (growth) of a player into the independent component due to the intrinsic capacity of this player and the dependent component resulting from the influence of this players by the other players [32]. The independent components and dependent components are coded as nodes and directed edges, respectively, into bidirectional, signed, and weighted (bDSW) networks. The integration of allometric scaling law into mODEs makes it possible to reconstruct informative, dynamic, omnidirectional, and personalized networks (idopNetworks) from any data domain, including inexpensive static data. Beyond correlation‐based and Bayesian networks, idopNetworks can capture a complete set of interaction properties, showing a broader impact on mechanistic modeling of complex systems [33, 34, 35, 36, 37, 38].

In this article, we implement the idopNetworks model to dissect the genomic mechanisms of plant grafting. We design an intra‐ and interspecific grafting experiment using young shoots of two distantly related poplar species from genus *Populus* species, *P. szechuanica* var. *tibetica* from section Tacamahaca, and *P. tomentosa* Carr. from section *Leuce*, reciprocally as the scion and rootstock. *P. szechuanica* var. *tibetica* is an ecologically important species, mainly distributed in southwestern China at altitudes from 2000 to 4500 m [39], whereas *P. tomentosa* Carr., widely distributed and cultivated across northern and eastern China, plays multiple roles in the forest industry and environmental conservation [40]. Because of their distant relatedness, the combination of favorable traits characterized by these two species cannot be made possible through sexual interspecific hybridization. Grafting is a proper approach for achieving this improvement goal. By analyzing transcriptomic data measured from the segments above and below the graft union, our unified computational‐experimental strategy can address the following questions of fundamental importance to successful grafting: (1) how each gene mediates scion‐rootstock crosstalk and how this mediation varies among different combinations? (2) How gene‐gene interactions mediate vascular reconnection? (3) How the rootstock influences the development of the shoot form, and how the scion impact the evolution of the rooting system? Our integrative computational‐experimental framework provides a unique approach for characterizing the systematic mechanisms mediating scion‐rootstock crosstalk via vascular reconnection.

## 2 Results

### 2.1 Compartment Index as a Predictor

**FIGURE 1:**
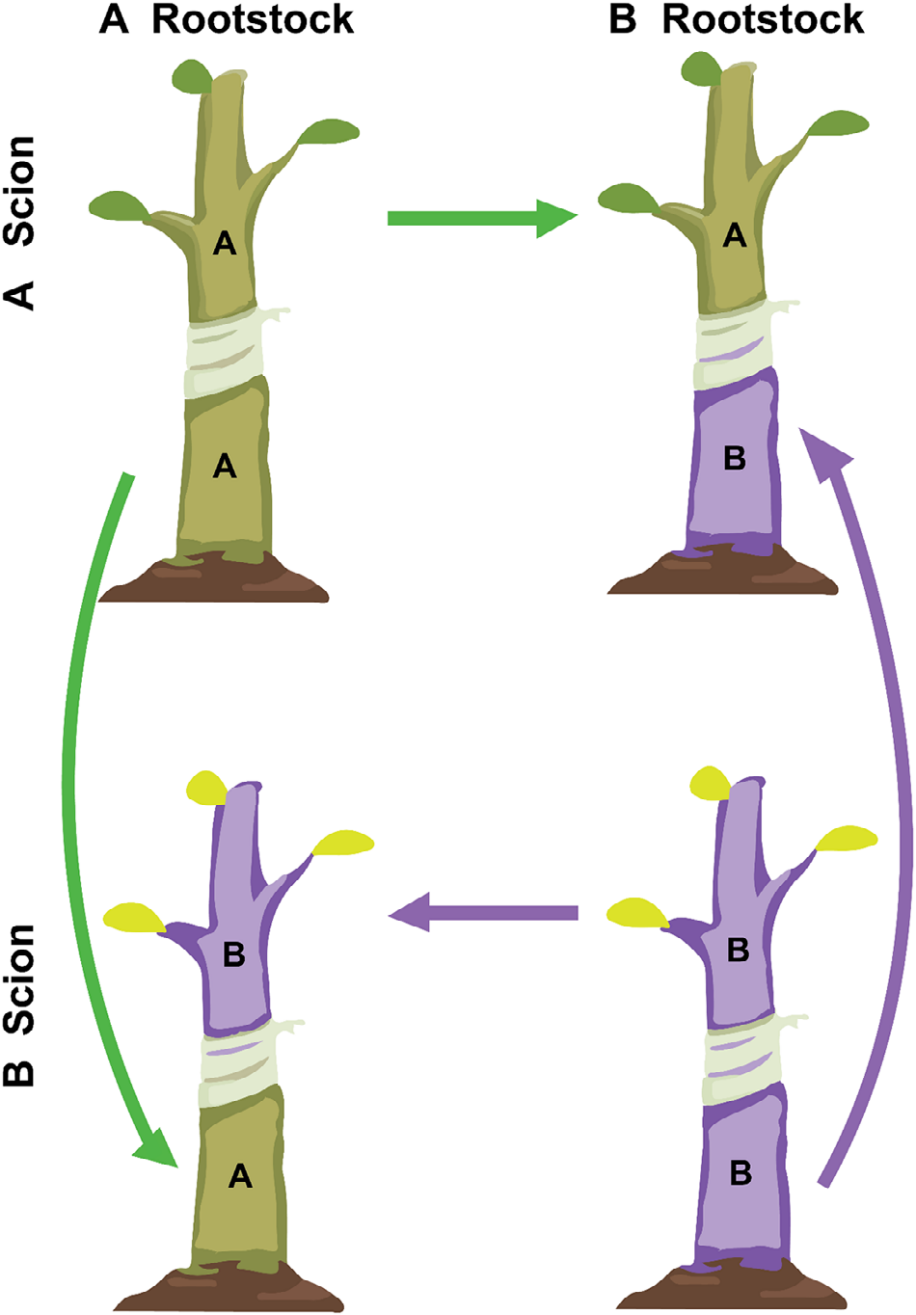
Experiment of reciprocal grafting between *Poulus szechuanica* var. tibetica (S) and *P. tomentosa* Carr. (T). (A) A grafted seedling of S/S, where the scion and rootstock of S are displayed. (B) A reciprocal grafting design involving S/S, S/T, T/S, and T/T. (C) Comparison in time trajectories of the compartment index expressed in a grafting segment linked to an alternative segment from different species. Gene expression was measured in the segments of boldfaced species abbreviations. (D) Change of compartment index with ecosystem index in the scions (blue) and rootstocks (red) of intraspecific (S/S and T/T) and interspecific (S/T and T/S) grafting combinations. Dots are values of mean compartment indices for all genes at eight time points, and thick lines are the goodness‐of‐fit of the compartment index by the power equation.

We produced the scions and rootstocks from juvenile trees of two distantly related poplar species, *P. szechuanica* var. *tibetica* (S) and *P. tomentosa* Carr. (T) and used these segments to self and reciprocally graft, producing four scion‐rootstock combinations, S/S and T/T (intraspecific grafting) and S/T and T/S (interspecific grafting) (Figure 1A,B). A grafted tree can be viewed as an ecosystem, composed of two compartments, a shooting scion and a rooting rootstock. For each combination, both the compartments from growing trees were profiled for thousands of genes at eight time points after grafting. For each grafting combination, we produce 21 344 genes from each replicate at each time point, forming a 21 344 × 24 data matrix. We calculate the sum of expression values of all genes from each compartment for each grafted tree and define this sum as the compartment index. The compartment index value reflects the carrying capacity of a compartment to respond to a grafting environment. We find that compartment index changes cyclically with time at initial stages of grafting for the scion and rootstock from each combination but they tend to be stable afterward, with the pattern of time trajectories depending on the segment sampled, species, and species combination (Figure 1C).

Total transcriptomic expression levels are markedly higher on the S segments than on the T segments, regardless of whether these segments serve as a scion or a rootstock and whether the scion‐rootstock combination is intraspecific or interspecific. By comparing the segments of a species grafted with its own segment and with the other species’ segment, we can characterize how interspecific grafting affects the expression pattern of genes. Overall, genes in the S scion are more strongly expressed when it is grafted to the S rootstock than to the T rootstock, implying that T inhibits gene expression of the S scion compartment. Yet, T does not affect gene expression of the S rootstock because compartment index values expressed in the S rootstock are similar in magnitude and time‐varying pattern when this rootstock is linked to the S scion and T scion. Compared to the T scion grafted to the T rootstock, total gene expression in the T scion grafted to the S rootstock is much more perturbed with time. A similar perturbation phenomenon is observed for the T rootstock linked to the S scion versus linked to the T scion. All this suggests that S interrupts the developmental stability of T’ gene expression. For the intraspecific grafting of S, higher compartment index values are detected on the scion compartment than on the rootstock compartment, whereas the inverse pattern holds for the intraspecific grafting of T.

We calculate the ecosystem index by summing the compartment index of two compartments for the same grafted tree and then plot the compartment index against the ecosystem index (Figure 1D). The range of ecosystem index among developmental times for interspecific grafts is much longer than that for intraspecific grafts. This suggests that interspecific adherence stimulates developmental fluctuations in gene expression. Also, the relative change of compartment index with ecosystem index varies dramatically between two compartments, with the pattern of this change heavily depending on the type of scion‐rootstock combination. Altogether from above, the time‐varying pattern of compartment index values from different grafting segments reflects some hidden genomic machineries of plant gratifying; a further investigation is needed to detail their mechanistic insight.

**FIGURE 2:**
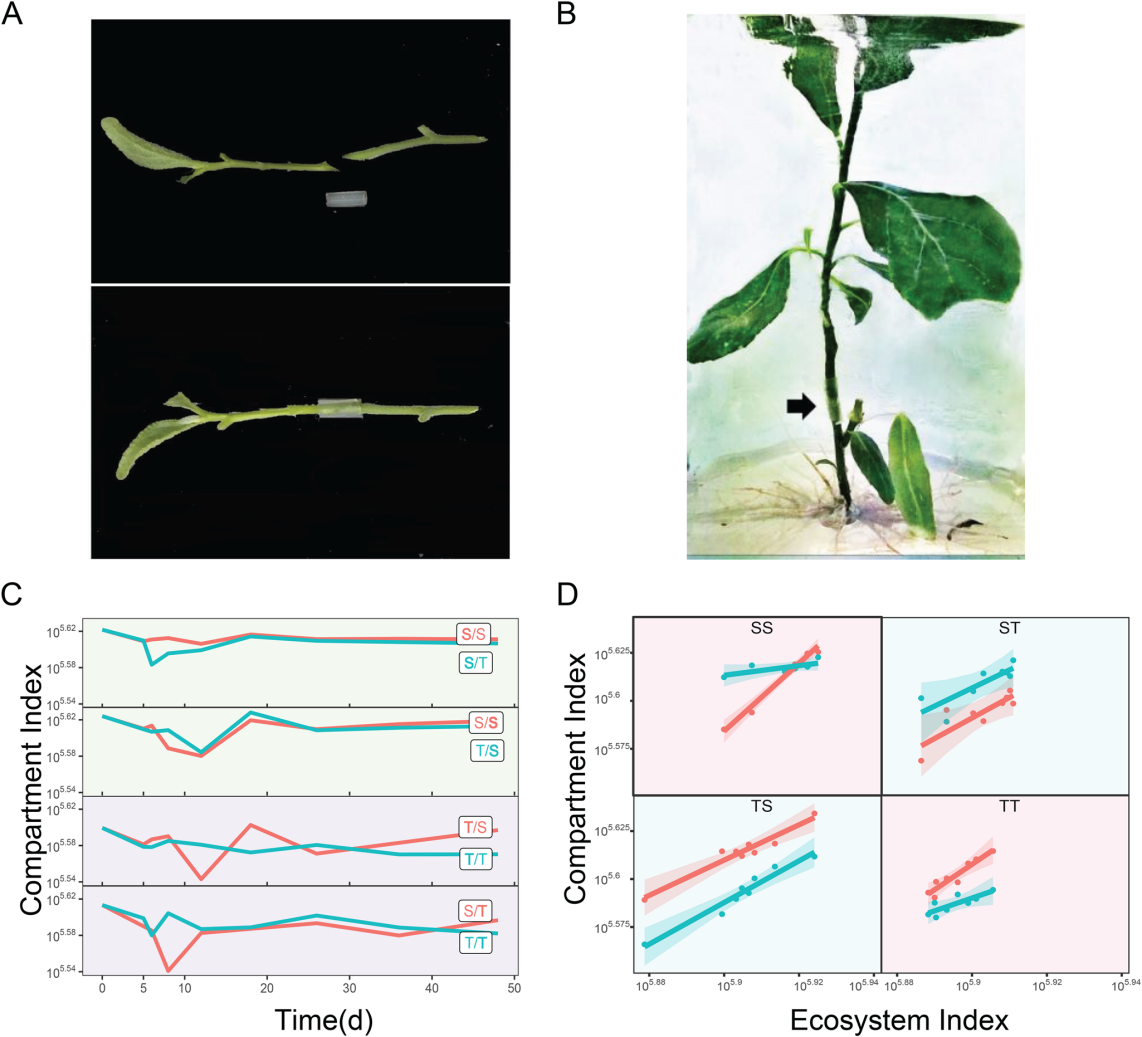
The profile change of eight randomly chosen genes with compartment index, expressed in the scion and rootstock of intraspecific (S/S and T/T) and interspecific (S/T and T/S) grafting combinations. Dots are expression values of a gene in each replicate at each of eight time points, and lines are fitted curves by the power equation.

The expression level of individual genes establishes a part‐whole relationship with the compartment index across samples, which obeys a physical rule that can be fitted by the allometric scaling power equation [41]. We find that the power fitting pattern of this relationship differs dramatically among grafting segments, scion‐rootstock combinations, genes, and species (Figure 2). MbContig44255 increases its expression with compartment index on the S rootstock under the S scion, but decreases its expression with compartment index on the T rootstock under the T scion. An inverse pattern is observed for the S scion of the S rootstock versus the T scion of the T rootstock. Pronounced differences in the compartment index‐varying expression pattern of MbContig44255 are also observed for the scion and rootstock segments of interspecific grafting. These differences are quite common for other genes (Figure 2). All these suggest that a complex mechanism exists to guide the grafting process.

### 2.2 Modeling Scion‐Rootstock Crosstalk

**FIGURE 3:**
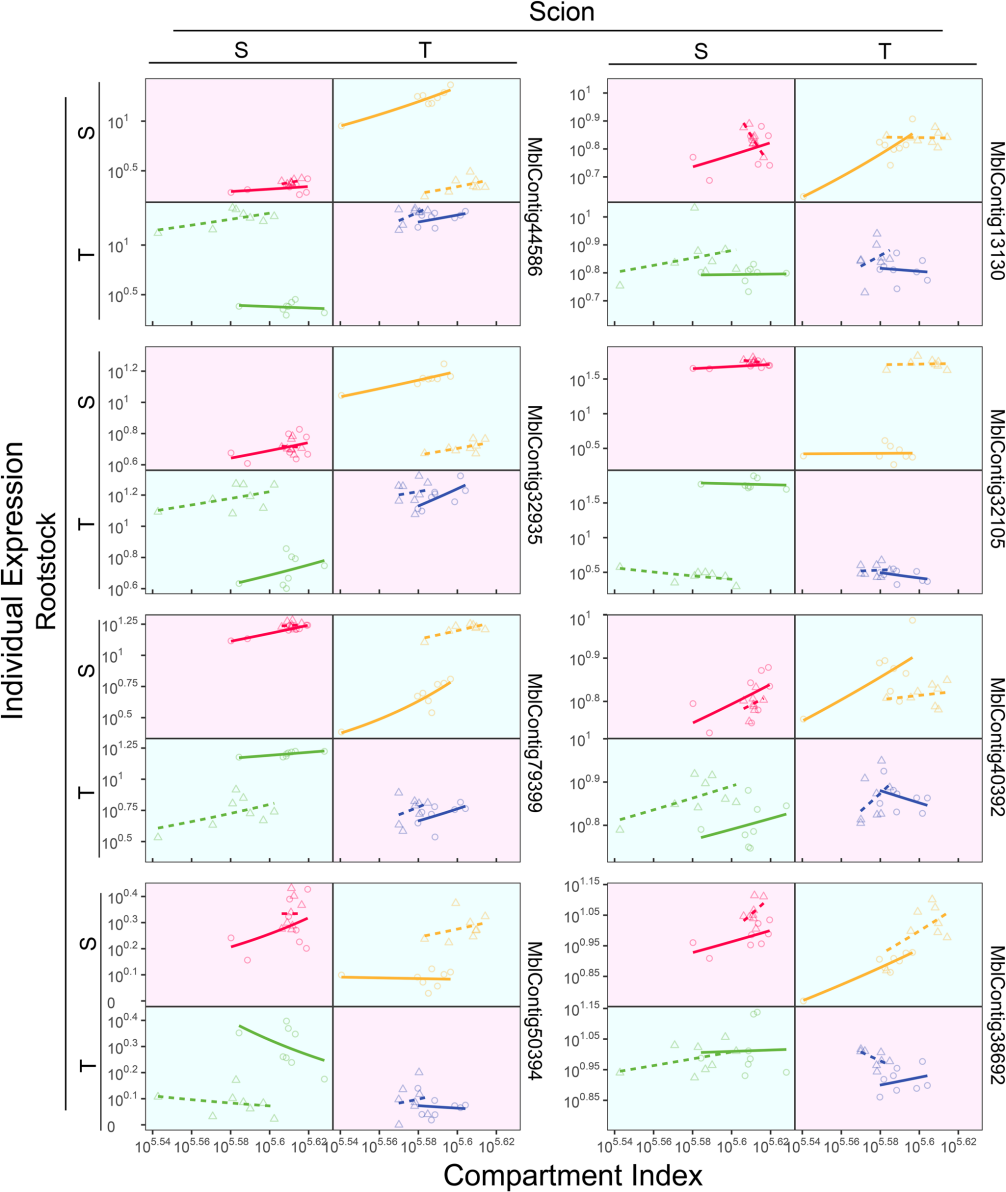
Crosstalk between the scion and rootstock induced by two representative genes in intra‐ (S/S and T/T) and interspecific (S/T and T/S) grafting combinations. The overall expression profiles of a gene (blue line) are decomposed into independent components (red lines) and dependent components (green lines).

For a grafted tree, the scion and rootstock grow as a whole, whose interactions can be modeled by game theory. Each segment behaves like a dove or hawk to interact with its linked counterpart, or it is neutral to its counterpart. In the Methods, we derive a pair of quasi‐dynamic mixed ordinary differential equations (qdMODEs) which specifies how a gene induces the reciprocal influence of one grafting segment on the other. In total, a gene may induce one of nine possible types of scion‐rootstock interactions during the growth of the grafted tree. Figure 3 illustrates the pattern of scion‐rootstock crosstalk induced by genes *MblContig1* and *MblContig44348* in four different intra‐ and interspecific grafting combinations. In the intraspecific grafting of species S, the scion acts as a dove to promote the expression of MbContig1 on the rootstock, but the rootstock acts as a hawk to inhibit the expression of this gene on the scion, forming a parasitic relationship (Figure 3 Top left). Yet, the scion and hawk cop‐exist peacefully without reciprocal influence on the expression of *MblContig1* in the intraspecific grafting of T. In interspecific grafting of S as the scion and T as the rootstock, strong parasitism is observed for the expression of *MblContig1*, with the T rootstock being promoted, whereas the S scion being inhibited. In interspecific grafting of T as the scion and S as the rootstock, the T scion inhibits the expression of *MblContig1* on the S rootstock, but the S rootstock has no effect on the T scion. Taken together, *MbContig1* activates the aggression of T to the grafted segment (including the scion and rootstock) of S, whereas this gene helps S, when it is used as the scion, to promote the rootstock.

*MblContig44348* mediates a predatory‐prey relationship for intraspecific grafting, where the scion behaves as a dove and the rootstock as a hawk for S, whereas the scion behaves as a hawk and the rootstock as a dove for T (Figure 3 Bottom). In interspecific grafting, the overall expression pattern of *MblContig44348* is quite similar between the scion and rootstock, but its underlying mechanism is segment‐dependent. We find that, induced by this gene, the scion is always altruistic to the rootstock in the interspecific grafting, but the rootstock derived from T tends to repress the scion from S, while the rootstock from S is neutral to the scion from T.

**FIGURE 4:**
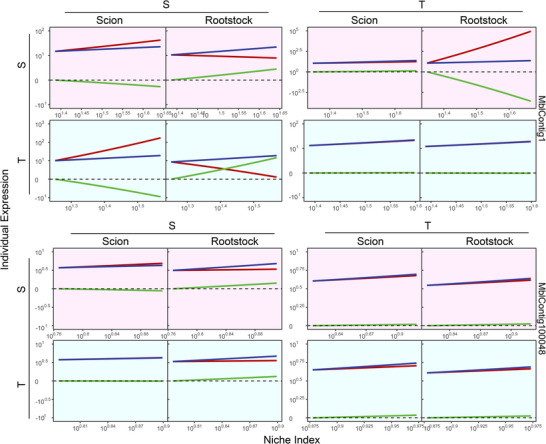
The frequency of genes mediating different types of ecological interactions during scion‐rootstock crosstalk for intraspecific (S/S and T/T) and interspecific (S/T and T/S) grafting combinations.

We calculate the distribution of genes that mediate different types of scion‐rootstock interactions in four grafting combinations (Figure 4). Many more genes are found to stimulate the cooperation between two grafting segments (both behaving like the dove) than their antagonism (both behaving like the hawk). Also, more genes are involved in shaping a commensalistic relationship (where one segment behaves like the dove while the other has no effect on the first) than an amensalistic relationship (where one segment behaves like the hawk while the other has an effect on the first). It is interesting to note that, among all types of interactions, the predatory‐prey relationships, in which the scion or rootstock behaves like a hawk and its counterpart behaves like a dove, are mediated by the largest number of genes. The number of genes mediating the same type of interaction varies, depending on the grafting type. The S activates more genes to mediate its scion as the dove to respond to the hawk rootstock than as the hawk to respond to the dove rootstock (Figure 4). In contrast, for T, more genes are activated to guide its scion as the hawk to respond to the dove rootstock than as the dove to respond to the hawk rootstock. This difference suggests that for a grafting plant, the S scion tends to help it to develop the root system, whereas the T scion displays a capacity to obtain help from the rootstock to develop the shoot system. Also, compared to the T scion, more genes from the S scion up‐regulate their expression in the hawk rootstock, whereas more genes from the T scion down‐regulate their expression in the dove rootstock than from the S scion. We find that while the S rootstock induces more genes that repress their expression in the dove scion than the T rootstock, the T rootstock induces more genes that promote their expression in the hawk scion than the S rootstock. All the above results together suggest that the overall gene expression of an interspecific grafting tree may benefit from the scion of S and the rootstock of T. Gene Ontology (GO) enrichment analysis illustrates the biological function of genes mediating various types of scion‐rootstock interactions for both intra‐ and interspecific grafting (Figure S1).

### 2.3 Reconstructing Multilayer and Multiplex idopNetworks for Plant Grafting

**FIGURE 5:**
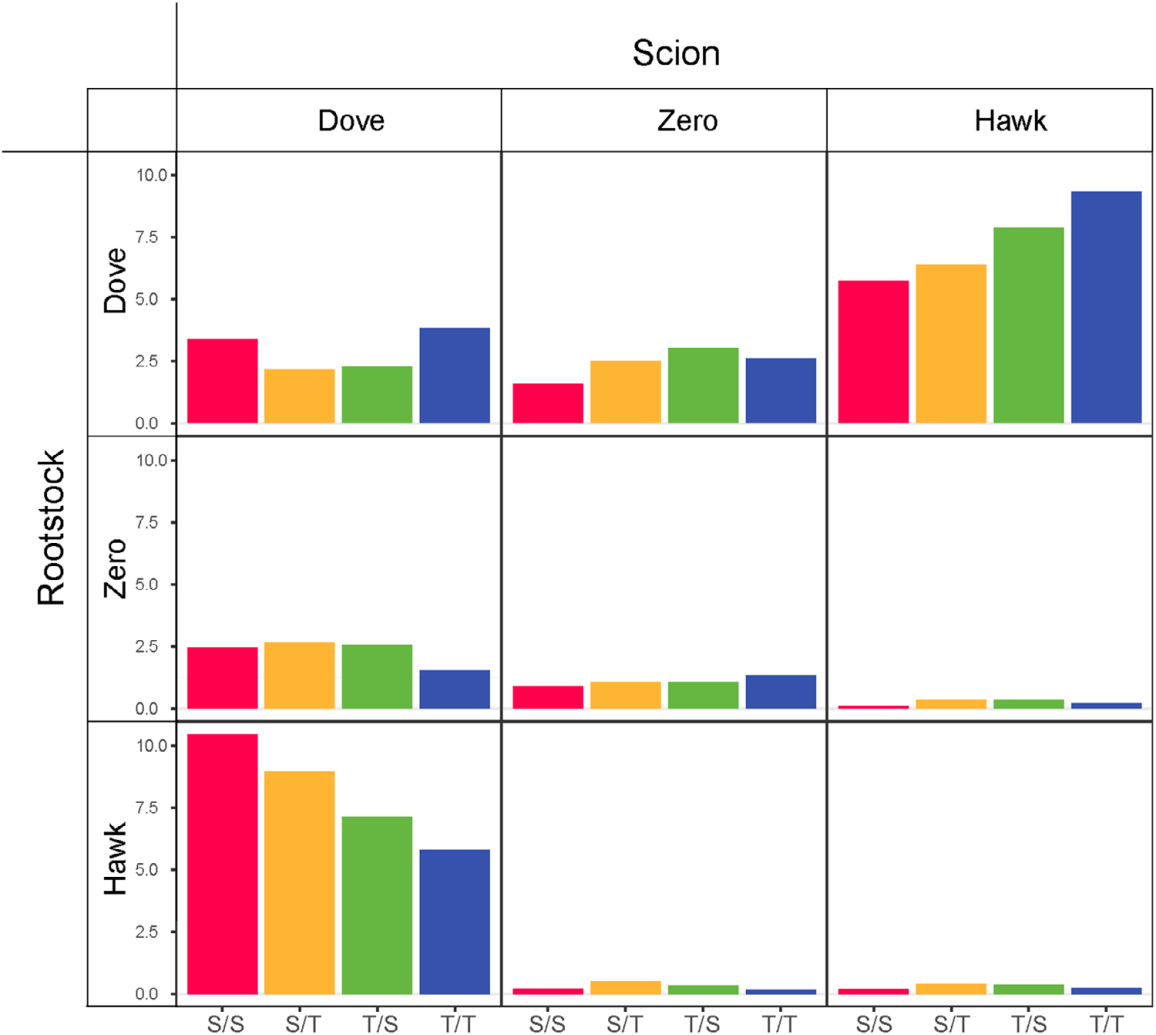
Octa‐variate functional clustering of genes into 33 modules M1 – M33 based on the similarity of compartment index‐varying gene expression trajectories across the scion segment (solid lines) and rootstock segment (broke lines) of intraspecific (S/S and T/T) and interspecific (S/T and T/S) grafting combinations. Numbers in parentheses are the number of genes within modules. Thick lines are the mean fitness of the power equation to all genes from the same module.

We implement octa‐variate functional clustering to classify 21 344 genes into 36 distinct modules (named M1‐M36) based on the similarity of their expression profiles over compartment index (Figure 5). These eight variables for joint classification include two intraspecific grafting combinations (S/S and T/T) and two interspecific grafting combinations (S/T and T/S), each unified with the scion and rootstock segments. Modules differ not only in the magnitude and compartment index‐varying pattern of gene expression, but also in the number of genes involved. For intraspecific grafting, some modules, such as M1, M2, M4, M10, M11, M15, and M35, display a greater mean gene expression level in both the scion and rootstock for S than for T, whereas for some modules, like M3, M5, M6, M8, M12, M20, M23, M26, M28, and M33, an inverse pattern is observed. The rest modules have no difference between the two species. For interspecific grafting, mean gene expression levels differ by grafting segment. Some modules, such as M1, have a larger mean gene expression level in the scion segment than in the rootstock segment, whereas some modules, such as M5, have an inverse pattern. There is also some module, like M18, which has no difference between the scion and rootstock segments. The 36 modules are the combination of these different patterns among intra‐ and interspecific grafting.

**FIGURE 6:**
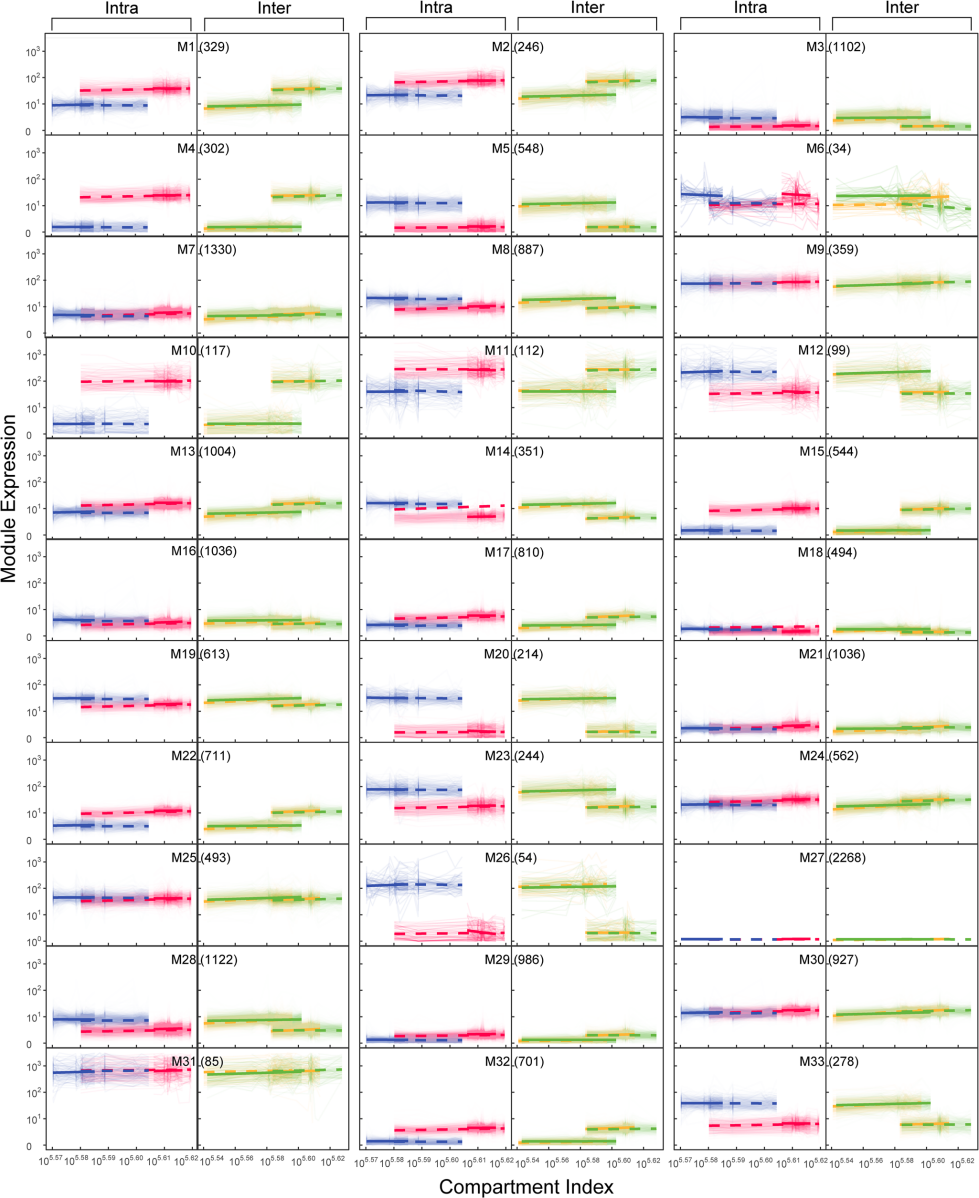
Network analysis expressed in the scion and rootstock of intraspecific (S/S and T/T) and interspecific (S/T and T/S) grafting combinations. (A) Coarse‐grained networks among 33 modules M1 – M33. Arrowed red and blue lines denote promotion and inhibition, respectively, with the thickness of lines proportional to the strength of interaction. (B) Centrality of the most important modules within the networks.

We further classify each module into distinct submodules by octa‐variate functional clustering and classify each submodule into distinct sub‐submodules. This process repeats until the number of genes within a unit a tractable level for network reconstruction. We reconstruct large‐scale interactome networks covering all genes for different grafting segments and scion‐rootstock combinations. These networks are multilayered, at the top of which is a coarse‐grained network among all modules (Figure 6A) and at the bottom of which are fine‐grained networks among individual genes. Modules M6 and M36 serve as hubs for all coarse‐grained networks, although S favors M6, whereas T favors M36 (Figure 6B). We find that the scion network and rootstock network in intraspecific grafting only slightly differ for S but markedly differ for T in terms of the type and number of hub modules. This suggests that S is more resilient to graft wounding than T. This can be confirmed by interspecific grafting, where S has a similar network structure when it is grafted to, or by, T. Yet, a pronounced change is observed in network structure for T when it is grafted to, or by, S.

**FIGURE 7:**
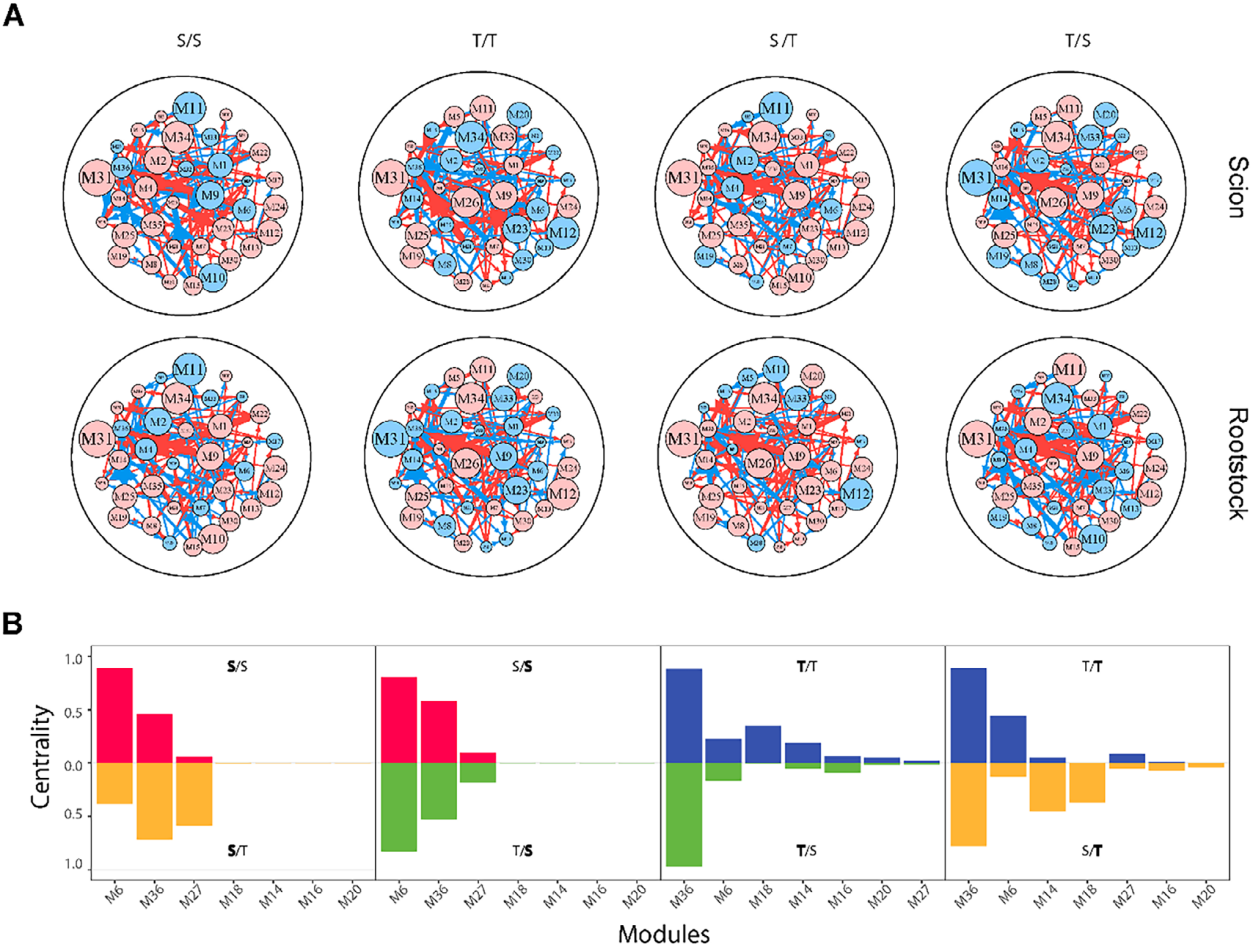
Network analysis expressed in the scion and rootstock of intraspecific (S/S and T/T) and interspecific (S/T and T/S) grafting combinations. (A) Networks among 20 submodules derived from module M36. (B) Fine‐grained networks among 80 genes from submodules SM36_16 of M36. Arrowed red and blue lines denote promotion and inhibition, respectively, with the thickness of lines proportional to the strength of interaction. (C) The decomposition of overall expression trajectories of two genes (blue lines) into independent components (red lines) and dependent components (green lines) due to the influence of other genes. ID of genes are indicated below.

We reconstruct fine‐grained interactome networks among individual genes derived from each module for different grafting segments and scion‐rootstock combinations. We use the “igraph” package in R to compute common network parameters [42], including mean distance, clustering coefficient, vertex connectivity, and diameter of multilayer regulatory networks (Table 1). We find that both the mean distance and clustering coefficient increase from coarse‐ to fine‐grained networks, suggesting the existence of a “small‐world” phenomenon at the bottom layer of the networks [43]. As an example, we illustrate the second‐layer networks among 20 submodules from module M36 (Figure 7A) and the fine‐grained networks among 80 genes derived from submodule SM36_16 that contains a candidate gene mediating plant grafting (Figure 7B). Within each fine‐grained network, we decompose the overall expression trajectories of each gene into its independent and dependent component trajectories (see Figure 7C for two examples). MblContig84328 is a candidate gene participating in the mediation of plant grafting (Histone‐lysine N‐methyltransferase CLF‐like), whose overall expression trajectory displays a similar pattern among four grafting combinations for each grafting segment. We find that this gene does not function in isolation rather than it is regulated by nine other genes. On the scion, the observed expression level of this gene is higher than its independent component because it receives a new positive influence by multiple genes. We find that MblContig84328 is, to a large extent, down‐regulated by MblContig84138 on the scion of interspecific grafting. By removing this negative regulation, the expression of MblContig84328 can be amplified, benefiting the success of interspecific grafting.

**TABLE 1:** Properties of multilayer regulatory networks.

	First Layer (Module)	Second Layer (Sub‐module)	Third Layer (Gene)
Mean Distance	2.717	2.051	4.342
Clustering coefficient	0.088	0.233	0.045
Vertex connectivity	0.355	0.450	0.144
Diameter	7.000	4.750	11.639

*Note*: In the network of the second layer and the third layer, each attribute adopts the mean value of all networks.

Other genes exhibit different patterns of expression across the scion and rootstock. MblContig23948 is more strongly expressed on the T scion than the S scion, as well as on the T rootstock than the S rootstock, for both intra‐ and interspecific grafting (Figure 7C), suggesting that this gene may play a critical role in shaping the growth of the grafting tree by regulating T‐specific expression. It is interesting to find that this gene is regulated by much fewer genes when it is expressed on the T grafting segment than on the S grafting segment. Yet, the strength of its regulation by regulators is strikingly stronger on the former than on the latter. All this suggests that the expression of MblContig23948 can be more readily manipulated on the T grafting segment than on the S grafting segment.

### 2.4 Hub Genes and Tracing Information Flow

**FIGURE 8:**
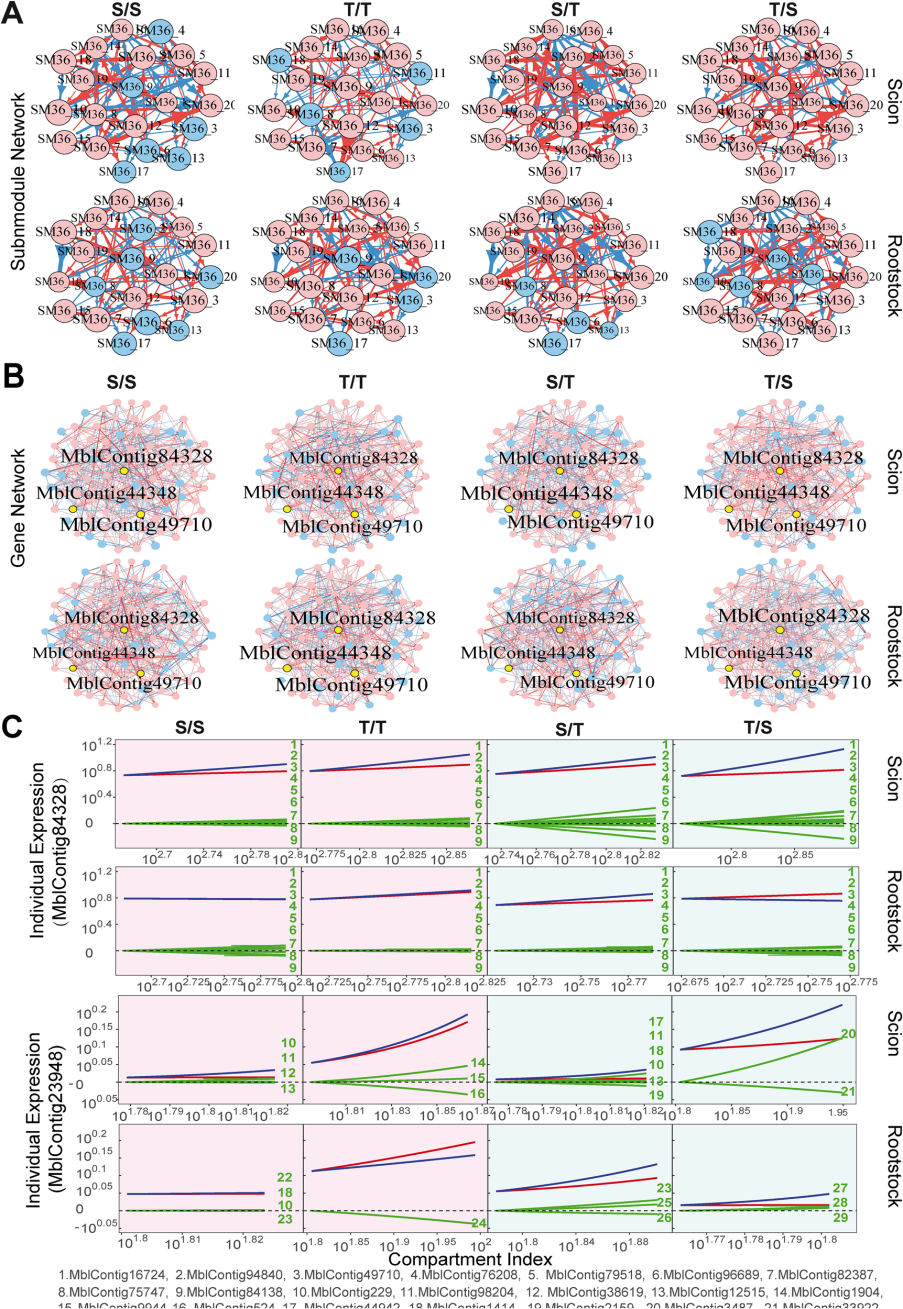
An overview of hub modules, GO enrichment, and its Sankey diagram. (A) The bubble plot of hub module M6 and M36, and only the top 10 terms are shown here. (B) The GRN among hub module M6 and submodule SM36_16, with hub genes heighted in yellow. (C) A Sankey diagram shows how regulatory effects flow in these networks, genes whose eigenvector centrality is greater than 0.5 is selected here. The red line and the blue line in the figure represent the promotion and inhibition effects, respectively, and the hub genes are highlighted by the red box.

A hub serves as the central point of connection within a network, allowing the system to communicate, propagate, and disperse information throughout all nodes. We perform a GO enrichment analysis to characterize the biological function of hub modules in the coarse‐grained network and hub genes in the fine‐grained networks (Figure 8A). We find that hub module M6 contains a large number of genes associated with auxin polar transport and auxin homeostasis, whereas numerous genes in hub module M36 are related to DNA‐binding transcription factor activity. By calculating the eigenvector centrality of each node in a network, we find that MblContig55294 is the hub gene of module M6, annotated as the Kinesin‐like protein KIN‐7D, chloroplastic. The hub sub‐module of module M36 is SM36_16, containing three genes of high eigenvector centrality, MblContig84328, MblContig44348, and MblContig49710, annotated as Histone‐lysine N‐methyltransferase CLF‐like, AP‐3 complex subunit mu‐like isoform X2, and Vacuolar protein sorting, respectively (Figure 8B). Hub submodules and hub genes for each module are listed in Table 2.

**TABLE 2:** Key submodules and genes of the three‐layer network.

Module	Submodule Number	Key Submodule	Key Gene
M1	NA		MblContig28164
M2	NA		MblContig78991, MblContig7560, MblContig66645, MblContig55452
M3	10	SM3_6	MblContig58829
M4	NA	NA	MblContig4769, MblContig88521
M5	12	SM5_2	MblContig63185
M6	NA	NA	MblContig55294
M7	17	SM7_1	MblContig29968
M8	11	SM8_2	MblContig87149
M9	NA	NA	MblContig21960
M10	NA	NA	MblContig103302
M11	NA	NA	MblContig83979, MblContig29599
M12	NA	NA	MblContig75370
M13	13	SM13_9	MblContig35867
M14	NA	NA	MblContig54858
M15	10	SM15_4	MblContig30016
M16	20	SM16_17	MblContig50998, MblContig3252, MblContig4534, MblContig1922
M17	20	SM17_4	MblContig6883, MblContig74957, MblContig20548
M18	10	SM18_9	MblContig78946, MblContig100227
M19	8	SM19_6	MblContig36130
M20	NA	NA	MblContig70680
M21	17	SM21_2	MblContig18946, MblContig1464
M22	14	SM22_2	MblContig63480
M23	NA	NA	MblContig46158
M24	18	SM24_8	MblContig30585
M25	8	SM25_5	MblContig48569, MblContig2371
M26	NA	NA	MblContig74116
M27	17	SM27_1	MblContig42967
M28	9	SM28_3	MblContig84357, MblContig56090
M29	18	SM29_2	MblContig48006
M30	13	SM30_1	MblContig38768, MblContig18206
M31	NA	NA	MblContig82173, MblContig48212
M32	12	SM32_1	MblContig94903, MblContig6156
M33	NA	NA	MblContig18601
M34	NA	NA	MblContig53375, MblContig48985
M35	NA	NA	MblContig17348
M36	20	SM36_16	MblContig44348, MblContig49710, MblContig84328

We examine the overall structure of grafting networks, identifying the small world phenomenon, i.e., a high degree of clustering, along with extensive global connectivity [44, 45]. We draw Sankey diagrams to trace the flow of information within the network, by considering a maximum number of six nodes as connected according to six degrees of separation. We analyze our findings by using the rootstock network of grafting combination S/T (Figure 8C). In module M6, hub genes MblContig55294 and MblContig42683 jointly up‐regulate the expression of gene MblContig14040. In submodule SM36_16, hub genes MblContig44348 and MblContig43936 together regulate the expression of MblContig11741, and while MblContig84328 is involved in regulating MblContig1535, MblContig49710 directly regulates MblContig11466, a gene as one of the ultimate regulatory targets for many genes and annotated as probable polygalacturonase isoform X2 involved in changes in cell wall morphology. In grapes, the activity of the enzyme encoded by this gene decreases after grafting [46], suggesting its relevance to grafting processes.

## 3 Discussion

In this article, we propose a computational‐experimental framework for understanding the genomic mechanisms of plant grafting, an important horticultural technique for improving plant productivity and stress resistance. The major advantages of this framework are twofold. First, we develop and implement a generalized statistical mechanics model for reconstructing gene regulatory networks mediating vascular reconnection during plant grafting. Networks have emerged as a potent instrument for identifying the interrelationships between various variables and investigating how these connections influence the inner mechanisms and dynamics of diverse physical, chemical, biological, and social systems [47]. Our network models are derived from the seamless integration of evolutionary game theory and graph theory through an allometric scaling law, which allows us to reconstruct informative, dynamic, omnidirectional, and personalized networks (idopNetworks) from static data. IdopNetworks are advantageous over traditional models by characterizing the genomic signature of scion‐rootstock crosstalk for the grafting plant and causal interrelations of gene expression between the grafting segments in large‐scale gene regulatory networks. Second, we design and conduct a grafting experiment, in which two different species are self‐ and reciprocally grafted to generate four scion‐rootstock combinations (see a similar reciprocal design for phenotypic plasticity research [48]). This experiment includes intra‐ and interspecific grafting and provides unique insight into asking, disseminating, and testing biologically meaningful hypotheses regarding the process of graft formation and regeneration.

Unlike previous studies, mostly focused on hormone crosstalk [49], our grafting modeling is data‐driven, as the first of its kind to quantify how exactly the scion and rootstock influence each other during the grafting process. In a grafting experiment using two *Populus* species, *P. szechuanica* var. *tibetica* (S) and *P. tomentosa* Carr. (T), We find that the manner in which the scion and rootstock crosstalk and communicate with each other is determined by specific genes, with the altruistic/predatory pattern of scion‐rootstock interactions involving more genes than the other patterns (Figure 4). It was observed that intraspecific grafting has a higher rate of survival (90%–95%) than interspecific grafting (75% for T/S and 55% for S/T) [50], which may be due to more genes that induce scion‐rootstock cooperation in intra‐ than interspecific grafting plants. It is interesting to note that for the S/S combination, more genes are involved in the promotion of the scion on the rootstock than the promotion of the rootstock on the scion, whereas the inverse pattern holds for T/T combination. For the S/T interspecific combination, the T rootstock inhibits the S scion, to a larger extent, by inducing more genes, than it promotes the S scion (S_dove/T_hawk > S_hawk/T_dove), whereas for the T/S interspecific combination, the S rootstock activates more genes to promote the T scion than to inhibit the T scion (T_hawk/S_dove > T_dove/S_hawk). This difference may explain why T/S grafting plants (where the scion is promoted) have a higher survival rate than S/T grafting plants (where the scion is inhibited) [50]. All this implies that a grafting tree can better grow when the scion is derived from T than from S and when the rootstock is derived from S than from T.

From a physiological perspective, the difference of survival rate between the two reciprocal interspecific combinations is likely due to lignification. Both S and T rootstocks were in vitro‐propagated from adventitious buds, but S exhibited lower lignification than T, which may facilitate the former's graft union formation. Previous studies in citrus showed that non‐lignified rootstocks have the highest survival (79.5%), followed by semi‐lignified rootstocks (26.7%) and fully lignified rootstocks (failing to survive) [51]. These results indicate that lower lignification promotes graft healing and success. In practice, by using it as a rootstock, the advantages of *P. szechuanica* var. *tibetica*, such as rapid growth, excellent wood quality, strong resilience, and rich genetic diversity [39], can be propagated. By using it as the scion, the strong resistance of *P. tomentosa* Carr. to many diseases, and insects, and drought can be maintained in grafting trees.

Plant grafting involves a complex gene regulatory network, but its reconstruction has proven to be difficult. To our best knowledge, we reconstruct one of the most advanced works – idopNetworks to characterize the genomic mechanisms of graft formation and regeneration. Our identification of hub genes by idopNetworks can be validated by comparing these genes with functionally characterized genes reported in existing grafting‐biology studies. In a fine‐grained gene network, MblContig84328 serves as a hub linking to many other genes to exert its function as an abscisic acid (ABA) response element. Widely involved in regulating plant grafting, ABA can induce cold resistance in seedlings’ grafted rootstocks and prevent water loss by restricting stomatal aperture [52]. In the grafting research of tomato wild type (WT) and the ABA‐deficient type *flacca*, the ABA content in the *flacca* scion is about twice that of the *flacca* rootstock [53]. Our model dissects the function of MblContig84328 into its independent and dependent components, better characterizing how this gene regulates scion‐rootstock crosstalk. MblContig44348, serving as a hub in the other fine‐grained gene network, is part of the AP‐3 complex related to the Golgi area and other peripheral structures. It can promote vesicle budding from the Golgi membrane and may directly participate in transportation to vacuoles, and also plays a role in maintaining lysosomal identity and regulating the transformation between storage and degradation vacuoles [54]. A third hub, MblContig49710, is annotated as a vacuolar protein sorting‐related protein, which participates in vesicular protein sorting. A study by Song et al. found that several protein bands exhibit a higher intensity on the grafted rootstock, with bands 11 and 17 involving a total of 20 proteins, including vacuolar protein sorting‐related proteins. These identified proteins may be induced by grafting a watermelon onto a gourd, playing biological roles in resisting biotic and abiotic stresses [55]. Module M6 serves as a major hub in the coarse‐grained networks, especially for S‐derived grafting segments. This module contains a hub gene MblContig55294, which mediates microtubule‐enhancing ATPase activity and regulates photosynthetic activity under different light conditions [56]. Many other hub genes can also be validated by previous functional studies. For example, MblContig11466 (annotated as polygalacturonase isoform X2 in grape), has been reported to mediate polygalacturonase (PG; EC 3.2.1.15), xylanase (XLN; EC 3.2.1.8), and cellulase (CEL; EC 3.2.1.4) in different grafted rootstocks [46]. As a hub gene, MblContig55294 was detected to be annotated with microtubule‐enhancing ATPase activity. Chaparro–Encinas et al. [56] reported that this gene is differentially expressed under temperature stress in durum wheat and may actively regulate photosynthetic activity under varying light conditions.

Taken together, we speculate that the grafting process in our poplar experiment involves complex coordination of various biochemical and molecular processes. First, grafting causes damage at the plant's graft interface. The cell walls between cells on both sides of the wound surface of the rootstock and scion change and may undergo hydrolysis. At the same time, the number of Golgi apparatus increases, widely secreting products, making it easier for the rootstock and scion to initially adhere [57, 58]. Subsequently, the isolation layer formed by grafting is broken through, and plasmodesmata are formed between the cells of the scion and the rootstock. Hormonal signal regulation is essential during poplar grafting, under the control of specific gene regulatory networks predominated by hub genes. These hub genes mediate hormone signal transduction, ion channels, transport proteins, and gene expression.

After grafting, plants undergo a series of biological changes. When genetic material is transferred to recipient tissues through the graft junction, it may play a crucial role within the plant for an extended period or might be metabolized and degraded. The abundance of genes could potentially influence the graft healing and regulatory processes. Our grafting design provides with a novel approach to dissect how the scion and rootstock interact with each other and characterize each gene's behavior in different tissues, together exploring the genomic mechanisms underlying rootstock‐scion crosstalk and the process of vascular reconnection for grafted plants. Also, our design gives a detailed guideline on how to increase the success rate of plant grafting. In general, because of close compatibility, intraspecific grafting is of greater success than interspecific grafting. Our design allows us to compare the difference between S/S and S/T as well as S/S and T/S in the way how genetic information transfers from the scion to the rootstock and vice versa. This information may provide a measure of increasing the rate of grafting success by altering gene expression in the T rootstock or the T scion, making it consistent to gene expression in the S rootstock and S scion, respectively. A similar measure can be made to increase the rate of interspecific grafting S/T and T/S by altering gene expression in the S scion and S rootstock, respectively.

The proper development of a grafted plant critically depends on the coordinated interactions between the scion and the rootstock that affect vascular tissue regeneration and eventually the union of the two plants [4]. We develop a computational‐experimental framework for dissecting the network dynamics over tissue using static RNA‐seq data. It can characterize the molecular machineries of plant grafting by creating a comprehensive atlas of how the scion and rootstock crosstalk over graft formation and regeneration, and how this crosstalk is mediated by gene regulatory networks. While this model provides a powerful means for horticultural biologists to understand the grafting process, it could be potentially be applied to uncover and quantify the foundational principles underlying organ transplantation in humans.

## 4 Methods

### 4.1 Experimental Design of Grafting

**FIGURE 9:**
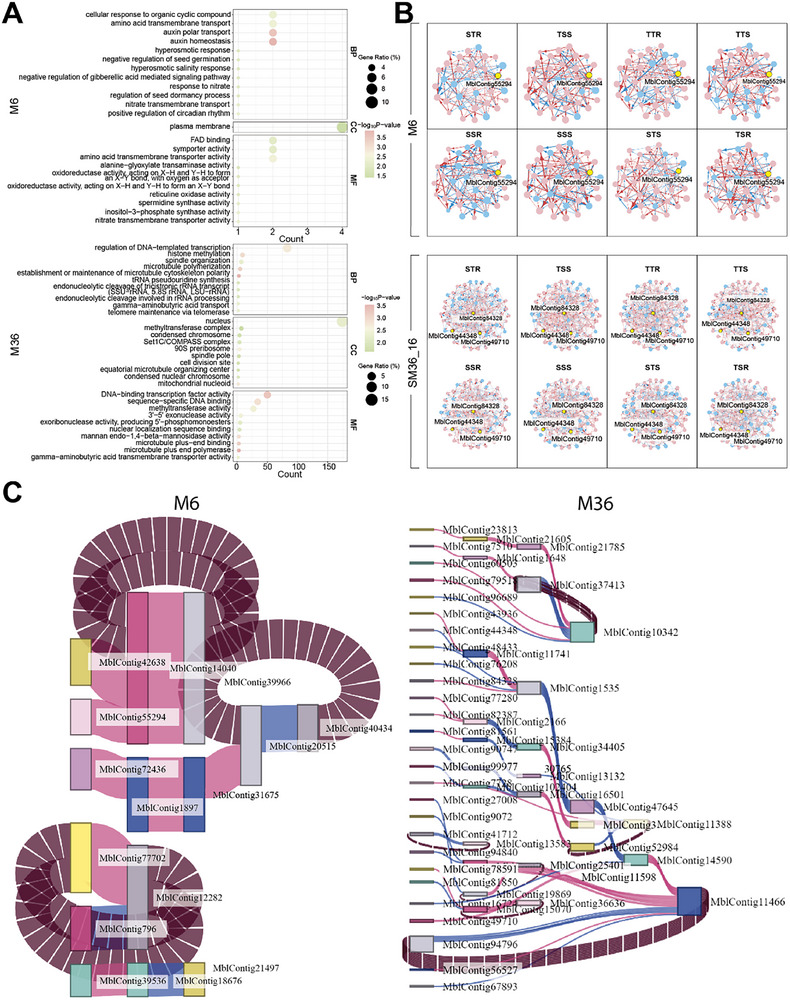
Scheme of plant grafting between two species A and B, which includes four combinations A/A. A/B, B/A, and B/B. Words at the left and right sides of the slash represent the scion and rootstock, respectively.

Suppose there are two species with distinct features. We aim to combine these features by grafting the scion of one species (A) on the rootstock of the other species (B). To study the genomic machineries of vascular reconnection that determine the success of plant grafting, we design an experiment of reciprocally grafting young tissues of the two species, leading to four scion‐rootstock combinations A/A, A/B, B/A, and B/B (Figure 9). The first two combinations are intraspecific grafts, whereas the last two are interspecific grafts. We profile the transcriptomic expression of genes at the scion and rootstock segments, which allow us to compare differences in gene expression between the scion and rootstock from each combination, between the scions of different species, and between the rootstocks of different species. All these differences are species‐ and grafting segment‐specific. Because of its high scion‐rootstock compatibility, intraspecific grafting has a greater likelihood of success than interspecific grafting [50], and, thereby, the differentiation of gene expression between inter‐ and intraspecific grafted plants can be used as a predictor of success/failure of plant grafting. Specific comparisons include: how genes are differentially expressed

- Between the A scion (S_A_) of inter‐ and intraspecific grafting.
- Between the B scion (S_B_) of inter‐ and intraspecific grafting.
- Between the A rootstock (R_A_) of inter‐ and intraspecific grafting.
- Between the B rootstock (R_B_) of inter‐ and intraspecific grafting.

The first and second comparison aims to test the capacity of species A and B to successfully graft (as a scion) on the other species, respectively, whereas the third and fourth comparison tests the capacity of species A and B to successfully be grafted (as a rootstock) on the other species, respectively.

Traditional transcriptomic analysis is to identify genes, their number, their regulation pattern, and their chromosomal distribution, which are associated with each of these questions (i) – (iv) involved in different aspects of plant grafting. However, such an analysis ignores how gene‐gene communication and interactions mediate vascular reconnection, limiting a detailed understanding of the genomic machineries for this grafting process.

### 4.2 Viewing a Grafting Plant as an Ecosystem

We view each grafted plant as a dynamic ecosystem composed of two interactive compartments, scion and rootstock. In each compartment, there are *m* transcript genes that interact with each other via cooperation or competition for a limited pool of ribosomes [59]. We define the sum of expression values of all genes from the scion or rootstock of a grafted plant as the compartment index and the sum of expression values of all genes from a whole grafted plant as the ecosystem index. The compartment index or ecosystem index represents a collective set of intrinsic and extrinsic conditions that facilitate the expression of genes in an ecological habitat. Let *y_ji_* denote the expression of a gene *j* from a compartment of grafted plant or sample *i* (*i* = 1, …, *n*). The compartment index of this sample is calculated as $E_{i}=\sum_{j=1}^{m}y_{ji}$, which represents the ability of this compartment to sustain the pool of ribosomes that are crucial for gene expression. The compartment index allows us to rearrange *n* samples in an ascending order (Table S1) so that *y_ji_* can be rewritten as *y_j_* (*E_i_*), expressed as a function of compartment index.

Since *y_j_* (*E_i_*) and *E_i_* are a part‐whole relationship, we introduce allometric scaling theory to describe this relationship using the power equation, expressed as

(1) $$y_{ji}\left(E_{i}\right)=\alpha_{j}E_{i}^{\beta_{j}}$$

where α_*j*_ is an intercept constant of the gene *j* and β_*j*_ correspond to its scaling exponent, which together determine the scaling shape of individual genes *j* with the compartment index. This power equation can also be used to describe the allometric relationship between compartment index and ecosystem index across samples.

### 4.3 Evolutionary Game Theory in Gene‐Gene Interactions

We hypothesize that gene‐gene interactions can be explained through the lens of evolutionary game theory [32]. According to game theory, a player decides to either cooperate or compete based on their strategy and that of their counterpart [60]. This theory is combined with evolutionary biology to leverage its application to interactions among nonrationality players [29]. We further implement the allometric scaling law to expand static evolutionary game theory into its quasi‐dynamic version to illustrate how strategies evolve in populations based on the compartment or ecosystem index. This quasi‐dynamic evolutionary game theory is combined with the prey‐predator theory to describe how the expression of a gene (as a prey) is affected by the expression of other genes (as a predator), through a set of qdMODEs with the derivative of compartment index (rather than the time derivative) [32], expressed as
(2)

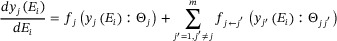

where the derivative of *y_j_* (*E_i_*) representing the expression level of a gene *j* (*j* = 1, …, *m*) from a compartment of the sample *i* (*i* = 1, …, *n*) is divided into two parts: the independent component *f_j_* (*y_j_* (*E_i_*): Θ_*j*_) which arises when the gene *j* is assumed to be independent from other genes and the dependent component 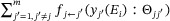 which emerges from the directional influence of the gene *j*′.

The independent and dependent components change with compartment index, but their mathematical forms are unknown. We implement a nonparametric approach, such as Legendre Orthogonal Polynomials (LOP), to fit the trajectories of these two components. Parameters Θ_*j*_ and 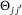 specifying the independent and dependent components, respectively, are the basis values of the trajectories corresponding to the LOP. Statistical algorithms can be developed to solve these qdMODEs, enabling the estimation of the independent and dependent components.

### 4.4 Regression Model and Variable Selection

Ample evidence shows that complex systems maintain dynamic stability against mutations, randomness, and environmental shifts through sparse connections [61, 62]. Thus, it is unlikely that each gene interacts with every other gene within the system. We implement variable selection to select a subset of the most influential genes interacting with a particular gene. Based on the property of LOP, the integral of Equation (2) does not change in basis parameters to be estimated, in which sense the integral is equivalent to the derivative. The integral of Equation (2) becomes an additive regression model, expressed as

(3)

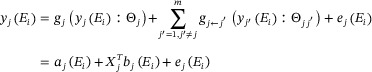

where *g_j_* (·) and *g*_*jj*′_(·) are the compartment index‐varying independent and dependent expression of a gene *j*, whose derivatives are *f_j_* (·) and *f*_*jj*′_(·) of Equation (2), respectively, and *e_j_* (*E_i_*) is the measurement error of gene *j* for the sample *i*, assumed to be independent and identically distributed with mean vector 0 and variance $\sigma_{j}^{2}$. Writing the integral as a regression model by letting *a_j_* (*E_i_*) =  *g_j_* (*y_j_* (*E_i_*): Θ_*j*_) and 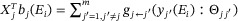 where $X_{j}^{T}$ is a vector with *m* − 1 units and *b_j_* (*E_i_*) is a vector of gene *j*'s dependent expression influenced by all genes, excluding the gene *j* itself.

For any given gene *j* considered as a response, there are *m* − 1 sets of dependent parameters indicating the regulatory influence of other genes on the given gene. To identify groups with non‐zero values, we employ Lasso and its variant method to select nonzero groups [63, 64]. The group Lasso estimation for dependent parameters can be represented as ${\dot{\beta }}_{i}=\left(\beta_{j|1},\text{…},\beta_{j|(j-1)},\beta_{j|(j+1)},\text{…},\beta_{j|d_{j}}\right)$, where *d_j_* (≪ m) signifies the count of the most influential genes interacting with the gene *j*. We perform variable selection by minimizing the following penalized weighted least‐squares criterion:

(4) $$\begin{array}{ccc} L_{1}\left({\dot{\beta }}_{i},\lambda_{j}\right) & = & {\left(y_{j}-a_{j}-X_{j}^{T}b_{j}\right)}^{T}Z_{j}\left(y_{j}-a_{j}-X_{j}^{T}b_{j}\right)\\ & & +\lambda_{1j}\sum_{j^{\prime }=1,j^{\prime }\neq j}^{m}{\left\|\beta_{j|j^{\prime }}\right\|}_{2} \end{array}$$

where $y_{j}=\left(y_{j}\left(E_{1}\right),\text{…},y_{j}\left(E_{n}\right)\right)$, $a_{j}=\left(a_{j}\left(E_{1}\right),\text{…},a_{j}\left(E_{n}\right)\right)$, $b_{j}=\left(b_{j}\left(E_{1}\right),\text{…},b_{j}\left(E_{n}\right)\right)$,  λ_1*j*_ is a penalty parameter determined either by BIC or the extended BIC, and $Z_{j}=\mathrm{diag}\left\{Z_{j}\left(E_{1}\right),\text{…},Z_{j}\left(E_{n}\right)\right\}$ to prescribe a non‐negative weight function within the range [*E*_1*j*_, E_*n*_], complying with the boundary conditions *Z_j_* (*E*_1_) = *Z_j_* (*E*_1_) = 0 to speed up the convergence rate.

### 4.5 Likelihood Approach for Network Reconstruction

After a small set of the most significant genes for each gene has been selected for each segment of each grafting combination (A/A, A/B, B/A, or B/B), we reduce an *m*‐dimensional system of full qdMODEs to an *m*‐dimensional system of sparse qdMODEs, expressed, in matrix notation, as

(5)

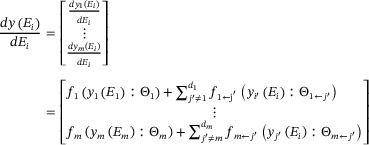

where the expression level of the gene *j* is determined by its independent expression components *f_j_* (·) and the cumulative dependent expression component $\sum_{j^{\prime }\neq j}^{d_{j}}f_{j\leftarrow j^{\prime }}\left(\cdot \right)$. Equation (5) represents the mathematical framework of Smith and Price's evolutionary game theory [29].

We derive a likelihood approach to estimate Equation (5). Let ϕ=(μ,Σ)∈Φ denote all model parameters and, thus, the likelihood function of **Φ** given gene expression data is written as

(6) $$L\left(\mu ;\Sigma \right)=f\left(y|\mu_{1},\text{…},\mu_{m};\Sigma \right)$$

where *y* is an *m* × *n* matrix containing all *y_j_*, *f*(·) is the *n*‐dimensional *m*‐variate normal distribution for *m* genes across *n* samples with the mean vector ***μ*** and covariance matrix **Σ**. By maximizing the likelihood in Equation (6), we can obtain the optimal solution for all model parameters. We implement the integrals of sparse qdMODEs in Equation (5) to model the mean vector and an autoregressive model to fit the structure of the covariance matrix and, therefore, the parameters to be estimated are those that fit the mean‐covariance structures. We apply the simplex algorithm to estimate those parameters under the maximization of the likelihood. The mathematical properties of qdMODE solutions have been proven by Griffin et al. (2020) [65].

After solving the sparse qdMODEs, we code the maximum likelihood estimates (MLEs) of the independent components as nodes and the MLEs of dependent components as edges into a gene regulatory network, expressed as a graph G  =  (V,  E) assuming pairwise interactions between genes, i.e.,

(7) $$\begin{array}{c} V=\left\{f_{1}\left(\cdot \right),\;f_{2}\left(\cdot \right),\text{…},\;f_{d_{m}}\left(\cdot \right)\right\}\\ E=\left\{f_{1\leftarrow 2}\left(\cdot \right),\text{…},\;f_{1\leftarrow d_{m}}\left(\cdot \right),\;R_{2\leftarrow 1}\left(\cdot \right),\text{…},R_{d_{m}\leftarrow 1}\left(\cdot \right)\right\} \end{array}$$

Such a network can capture the three distinct properties of gene interactions: bidirectional influence, the intensity of interaction, and the nature of interaction (promoting or inhibiting). This is because the value of $f_{j\leftarrow j^{\prime }}\left(\cdot \right)$ defines the magnitude and direction (positive or negative) of the influence of *j*′ on gene *j*. Compared to many existing networks that fail to simultaneously capture these properties, G  =  (V,  E) is regarded as being fully informative [34].

### 4.6 Significance Test of Gene‐Gene Interactions

A significant challenge in network reconstruction is how to determine the statistical significance of edges representing the associations between nodes. Here, we propose a likelihood ratio method for interaction testing. The null hypothesis is given by all genes that are expressed independently, and the expression change rate for each gene can be depicted using a simplified quasi‐dynamic ODE system, written as

(8) $$\frac{dy_{j}\left(E_{i}\right)}{dE_{i}}=f_{j}\left(y_{j}\left(E_{i}\right):\Theta_{j}\right)$$

which corresponds to the alternative hypothesis that there is at least one significant gene interaction within the network. We have obtained the likelihood values of ${\hat{L}}_{1}$ through Equation (6), and the likelihood value ${\hat{L}}_{0}$ of Equation (8) can be calculated accordingly. We further estimate the log‐likelihood ratio (LR) to test if interactions exist, expressed as

(9) $$\mathrm{LR}=-2\log\left({\hat{L}}_{0}/{\hat{L}}_{1}\right)$$

By reshuffling the expression data across *n* samples, we calculate the LR value. Repeating this permutation process 1000 times, we can determine the 95th percentile from these LR values, which serves as a critical threshold.

### 4.7 Competition/Cooperation and Hub Node Detection

We have described the statistical procedure for solving qdMODEs to obtain the MLEs of Θ_*j*_ and $\Theta_{j\leftarrow j^{\prime }}$. Based on these estimates, we can actually calculate the expression values of independent expression components and dependent expression components. The MLEs of these two components offer insights into how these genes behavior and interact with each other during the grafting process. For example, if the value of *f*_1 ← 2_(·) is positive, and the value of *f*_2 ← 1_(·) is positive, then this suggests that genes 1 and 2 are mutualistic, i.e., they promote each other and form a cooperative relationship. All possible types of gene‐gene interactions detected by our computational model are summarized in Table S2.

Within a network, a node with considerably more connections than the average is termed a hub, and such hubs are often perceived as pivotal in influencing the network's structure [66]. While these hubs form the backbone of a network, nodes on the network's outskirts may collaboratively contribute to the network's overarching design [67]. We introduce the idea of hubness by assuming that any node in a network has the potential to act as a hub, though the degree varies. We use eigenvector centrality to measure the hubness of each node, which better emphasizes the value of neighboring nodes when compared with other indicators.

### 4.8 Reconstructing Multilayer and Multiplex Networks Through Functional Clustering

In practice, the number of genes is often too large for direct network reconstruction. According to the developmental modularity theory, a complex system can typically be divided into multiple distinct network communities, within which the components are interconnected more closely compared to those from other communities [68, 69]. To detect these network communities, we implement function clustering [70] to divide all *m* genes into distinct modules according to the similarity of their compartment index‐varying expression trajectories. This clustering approach enables us to break down a large network into several interconnected smaller networks, addressing the challenge of network dimensionality. Let $y_{j}=\left(y_{j1},y_{j2},\text{…},y_{jn}\right)$ denote the expression of a gene *j* across all samples. We formulate a mixture‐based likelihood model for functional clustering, expressed as

(10) $$L\left(Y|\Theta \right)=\sum_{j=1}^{m}\ln\left(\sum_{k=1}^{K}\pi_{k}N\left(y_{j}|\mu_{k},\Sigma \right)\right)$$

where *Y* is an *m* × *n* matrix containing all ***y**_j_*, *K* is the number of modules, π_*k*_ is a prior probability indicating the proportion of the *k*th module ($\sum_{k=1}^{K}\pi_{k}=1$), and N(·) is the *n*‐dimensional multivariate normal distribution with mean vector ***μ**_k_* for module *k* and covariance matrix **Σ**. In practice, we implement the power Equation (1) to model the mean vector for each module and the autoregressive model, such as the first‐order structured ante‐dependence (SAD(1)) model, to fit the residual covariances of gene expression among different compartment indices [61].

We use a combination of the Expectation‐Maximization (EM) algorithm and the simplex algorithm to estimate the parameters that model the mean vector for each module and the covariance matrix. The optimal number of mixture components (or modules) is identified according to the Bayesian information criterion (BIC). After all parameters are estimated, we calculate the posterior probability (ω_*j*,*k*_) of a gene that is allocated to a specific module using the following formulas:

(11) $$\omega_{j,k}=\frac{\pi_{k}N\left(y_{j}|\mu_{k},\Sigma \right)}{\sum_{k^{\prime }=1}^{K}\pi_{k^{\prime }}N\left(y_{j^{\prime }}|\mu_{k^{\prime }},\Sigma \right)}$$

By calculating the posterior probabilities of a gene that belongs to each of the modules, we assign this gene to an optimal module that has the highest posterior probability.

If the number of genes in a certain module still contains too large, we further subdivide it through another round of functional clustering. In the context of network theory, this traceable number aligns with Dunbar's law, often cited when reconstructing networks, especially in the realm of social networks [71, 72].

Equation (10) presents a one‐variate functional clustering procedure, which can be extended to include all segments in four scion‐rootstock combinations. In this case, an eight‐variate functional clustering procedure is formulated. It is reasonable to assume that while the scion and rootstock segments are dependent for the same grafted plant, these can be regarded as being independent among different grafted plants. Under this assumption, the residual covariance matrix is partoitioned into the following four blocks:

(12) $$\begin{array}{ccc} & & \Sigma_{\mathrm{AA}}=\left[\begin{array}{cc} \Sigma_{\mathrm{AA}}^{S} & \Sigma_{\mathrm{AA}}^{\mathrm{SR}}\\ \Sigma_{\mathrm{AA}}^{\mathrm{SR}} & \Sigma_{\mathrm{AA}}^{R} \end{array}\right],\;\;\Sigma_{\mathrm{AB}}=\left[\begin{array}{cc} \Sigma_{\mathrm{AB}}^{S} & \Sigma_{\mathrm{AB}}^{\mathrm{SR}}\\ \Sigma_{\mathrm{AB}}^{\mathrm{SR}} & \Sigma_{\mathrm{AB}}^{R} \end{array}\right],\;\;\Sigma_{\mathrm{BA}}=\left[\begin{array}{cc} \Sigma_{\mathrm{BA}}^{S} & \Sigma_{\mathrm{BA}}^{\mathrm{SR}}\\ \Sigma_{\mathrm{BA}}^{\mathrm{SR}} & \Sigma_{\mathrm{BA}}^{R} \end{array}\right],\\ & & \;\;\Sigma_{\mathrm{BB}}=\left[\begin{array}{cc} \Sigma_{\mathrm{BB}}^{S} & \Sigma_{\mathrm{BB}}^{\mathrm{SR}}\\ \Sigma_{\mathrm{BB}}^{\mathrm{SR}} & \Sigma_{\mathrm{BB}}^{R} \end{array}\right] \end{array}$$

which are the covariance matrix blocks between the scion (S) and rootstock (R) from the same combination A/A, A/B, B/A or B/B. Each block can be modeled by SAD(1) [73].

### 4.9 Characterizing How Scion‐Rootstock Crosstalk Occurs

The scion and rootstock crosstalk within the grafted plant through genes. Coordinated inter‐organ crosstalk is crucial for the success of plant grafting by maintaining various resources for the transportation of biomolecules between the scion and rootstock [74]. We use qdMODEs to model how a gene induces such scion‐stock crosstalk. Let *y_jl_* denote the expression value of a gene *j* on compartment l (*l* = 1 for rootstock, 2 for scion). From this perspective, the scion and rootstock can be viewed as an ecosystem with a shared environment. We calculate the sum of abundance of gene *j* over the two segments from a sample *i* (*N_i_*) and define it as the niche index of this gene in this sample (Table S1). Like Equation (3), we build two coupled qdMODEs as

(3)

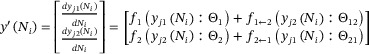

where the first and second terms of the equation at the right‐hand side are independent and dependent components, respectively. These two components can be smoothened using a nonparametric method such as LOP [75]. By estimating the dependent components, we can characterize how a gene induces cooperation or competition between the scion and rootstock. If both *f*_1 ← 2_(*y*_*j*2_(*N_i_*): Θ_12_) and *f*_2 ← 1_(*y*_*j*1_(*N_i_*): Θ_21_) are positive, then this suggests that the scion and rootstock promote each other (mutualism) through gene *j*. On contrast, the two segments inhibit each other (antagonism) through this gene if both *f*_1 ← 2_(*y*_*j*2_(*N_i_*): Θ_12_) and *f*_2 ← 1_(*y*_*j*1_(*N_i_*): Θ_21_) are negative. If one dependent component is positive, whereas the other is negative, the scion and rootstock establish an altruistic/predatory relationship through gene *j*. In some cases, one dependent component is positive or negative, but the second has no effect on the first, which implies commensalism or amensalism that drives scion‐rootstock crosstalk by this gene.

### 4.10 Plant Material and Experimental Design

We conducted a micro‐grafting experiment by reciprocally grafting young scions to young rootstocks between two *Populus* species, *P. szechuanica* var. *tibetica* (S) and *P. tomentosa* Carr. (T) from two different sections of the *Populus* genera, to form four grafting combinations, S/S, S/T, T/S, and T/T. To remove age effects, we produced young seedlings by inducting callus redifferentiation under the same conditions. The seedlings were cut at a 30‐degree angle and connected using a silicone tube, ensuring a maximum wound contact and a secure bond (Figure 1A). Each combination has 24 replicates, allowing destructive sampling at different stages after grafting. We used Carboxyfluorescein Diacetate (CFDA) fluorescence to trace phloem and xylem reconstruction at the graft union, confirming a successful connection between the rootstock and scion. Stem segments from both the scion and rootstock, approximately 1 cm above and below the graft union, were collected for RNA extraction and transcriptomic measurements at 8 time points, 6, 8, 12, 18, 26, 36, and 48 days after grafting (DAG). For each sample, tissues from six biological replicates were pooled to reduce experimental variability. Measurements at each time point were based on three clonal replicates, with each replicate including a mixture of 6 samples from multiple stem positions to minimize the influence of individual differences. The samples taken were immediately frozen in liquid nitrogen after collection and stored at −80 °C. These samples are named after scion species‐rootstock species‐sample position‐sampling time, e.g., “STR38” represents “the rootstock of *P. szechuanica* var. *tibetica* /*P. tomentosa*, the 3rd replicate, 8 DAG”.

### 4.11 RNA‐Seq and Data Processing

The total RNA of the samples was extracted with the Plant Total RNA Extraction Kit (DP432, Tiangen, China) according to the manufacturer's instructions. After agarose gel electrophoresis and spectrophotometer detection (NanoDrop 2000, Thermo Fisher, USA), qualified RNA samples were then sequenced on the Illumina HiSeq XTen platform with pair‐end 150 bp (PE150) by Novogene Co, Ltd. (Beijing, China).

In order to obtain a high‐quality dataset, adapters of sequences were cut, and low‐quality reads were removed using FastQC (v0.11.9), and then the clean reads of *P. szechuanica* var. *tibetica* and *P. tomentosa* were assembled using Trinity (v2.8.5) with default parameters, respectively. GICL (release date 2010‐07‐22) was used to cluster the assembled transcripts so as to further remove redundancy. Subsequently, the clustering result were step as a reference sequence, use RSEM and call Bowtie2 (v2.4.0) to map the clean reads to the transcript, and gene expression levels were determined using the FPKM (fragments per kb per million mapped fragments) method by RSEM (v1.3.3). To merge the expression matrix of two poplars, BLAST (v2.11.0) was used to screen out the sequences with similarity greater than 99% and length greater than 300 bp. In addition, sva package (v3.14) was used to remove potential batch effects, and the mean values of three repetition were taken to generate the final expression matrix data. Meanwhile, functional annotation of each sequence data was adopted by Blast2GO (v5.2) to get the Gene Ontology annotations.

## Conflicts of Interest

The authors declare no conflicts of interest.

## Supporting information




**Supporting File**: advs73699‐sup‐0001‐SuppMat.png.

## Data Availability

The data that support the findings of this study are available from the corresponding author upon reasonable request.
