# Supplementary figures and images for: A Statistical Mechanics Model to Decode Tissue Crosstalk During Graft Formation
Source: Adv Sci (Weinh). 2026 Jan 7;13(10):e23373. doi: 10.1002/advs.202523373 (PMC12915093; doi:10.1002/advs.202523373)

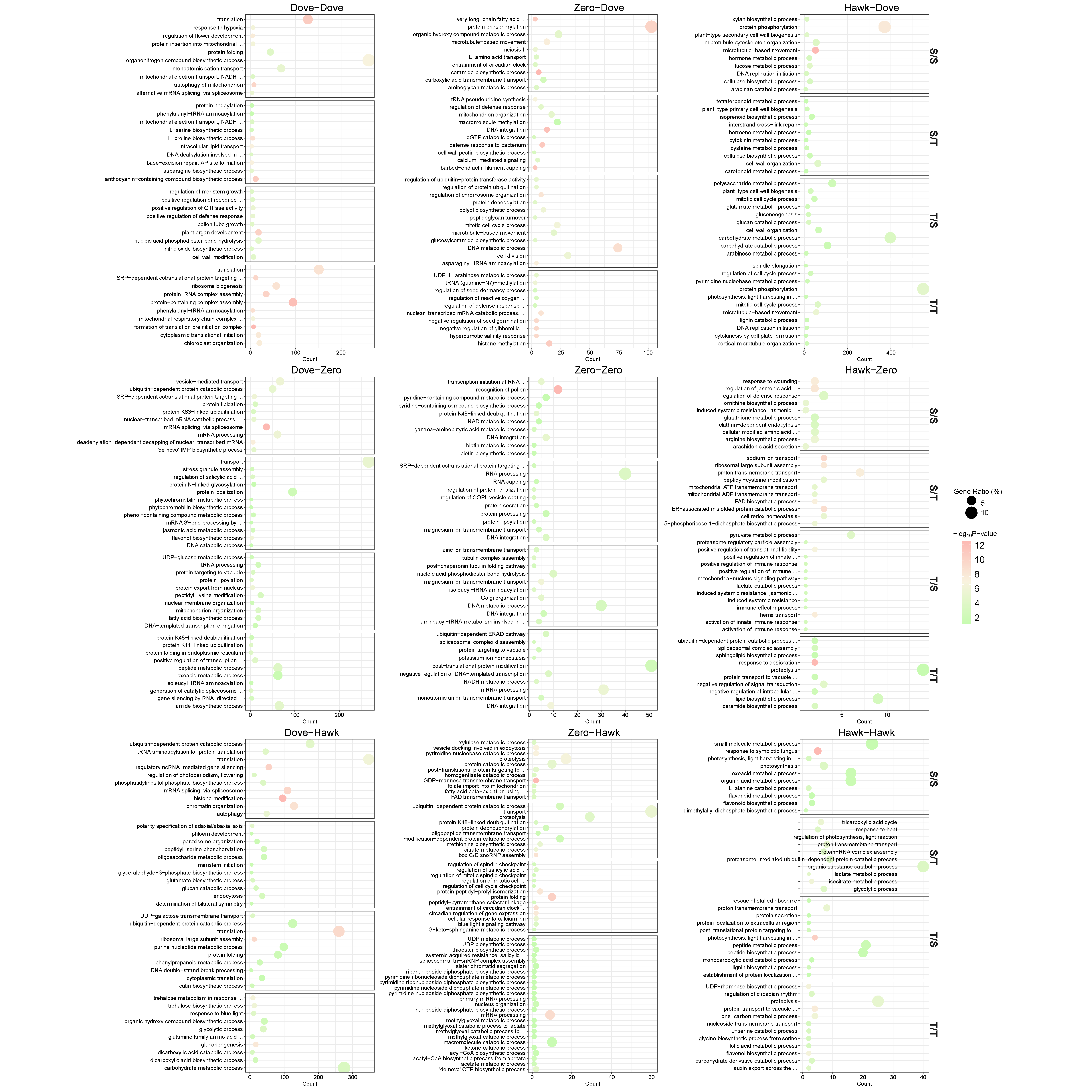

Supplement: Supplementary file 1 — Supporting File: advs73699‐sup‐0001‐SuppMat.png. [file ADVS-13-e23373-s001.png]
